# Idiopathic chylopericardium temporally associated with short-term high-fat dietary excess in a young man: a case report

**DOI:** 10.3389/fcvm.2026.1863669

**Published:** 2026-10-08

**Authors:** Lianjing Liang, Shitong Su, Peng Yao, Yu Cao

**Affiliations:** 1 Department of Emergency Medicine and Institute of Disaster Medicine, Institute of Emergency Medicine, West China School of Medicine, West China Hospital, Sichuan University, Chengdu, China; 2 Department of Pathology, West China Second University Hospital, Sichuan University, Chengdu, China

**Keywords:** conservative treatment, high-fat dietary, hypertriglyceridemia, idiopathic chylopericardium, pericardial effusion

## Abstract

Idiopathic chylopericardium is a rare and potentially serious condition typically managed conservatively, though surgical intervention is often required. This case report describes a 22-year-old man with no significant past medical history who presented with acute chest pain and dyspnea following a one-week period of excessive high-fat dietary intake. Physical examination revealed a characteristic forward-leaning posture. Laboratory evaluation demonstrated hypertriglyceridemia, while imaging confirmed moderate pericardial effusion without tamponade or constriction. Pericardiocentesis yielded 350 mL of chylous fluid, and subsequent lymphoscintigraphy suggested cessation of radiotracer transit at the supradiaphragmatic level. Comprehensive serologic testing ruled out infectious, autoimmune, and neoplastic etiologies. Following a presumptive diagnosis of idiopathic chylopericardium, the patient was successfully treated with a fat-restricted diet and octreotide, leading to complete symptom resolution and no recurrence at 2-month follow-up. This case highlights a temporal association between an extreme short-term high-fat dietary intake and the subsequent development of idiopathic chylopericardium. However, causality cannot be established based on a single case, and the current evidence supports only an association rather than a causal relationship, and supports the efficacy of conservative treatment in selected patients without hemodynamic compromise.

## Introduction

Chylopericardium represents an infrequent clinical entity, most frequently attributed to preceding cardiothoracic procedures, mediastinal neoplastic processes, and thoracic traumatic injuries. Idiopathic chylopericardium occurs considerably less common (1, 2). Idiopathic chylopericardium is a benign condition; however, in some cases, it is a life-threatening disorder, such as causing cardiac tamponade (3) and constrictive pericarditis (4). Conservative management has a reported failure rate ranging from 57% to 67% (1, 3), and a recent systematic review of 45 patients found that pericardiocentesis alone was frequently followed by recurrence or need for further intervention (2). This case suggests that extreme short-term high-fat dietary intake may serve as a temporal trigger for idiopathic chylopericardium in young adults, and that conservative management with fat restriction and octreotide can be effective in hemodynamically stable patients.

## Case presentation

A 22-year-old man came to our facility with acute and continued chest pain and shortness of breath over the past 4 h. Physical examination showed the patient to be in good health with a resting heart rate of 79 bpm, and a lying down blood pressure of 118/69 mm Hg. The patient was also having a characteristic forward leaning posture, but no other abnormalities were identified on the physical assessment. Interestingly, he reported a marked change in dietary and lifestyle habits in the 10 days prior to symptom onset. Specifically, he had consumed a self-described ‘excessively high-fat and high-calorie’ diet continuously for one week. However, the dietary intake was not quantified, and this temporal relationship does not imply causation. Peripheral blood test showed triglyceride levels over 3.17 mmol/L (Normal range: 0.29–1.70 mmol/L) and cholesterol of 4.46 mmol/L (Normal range: 2.80–5.17 mmol/L). He was not taking any regular medications. In addition, he reported no family history of hyperlipidemia and cardiovascular conditions.

Electrocardiogram showed low-voltage complexes and sinus arrhythmia (Figure 1A). Emergency transthoracic echocardiography was performed to assess the pericardial effusion and rule out cardiac tamponade. A moderately large pericardial effusion was quantified as follows: posterior pericardial space, 23 mm; apical, 16 mm; left lateral, 21 mm; right lateral, 11 mm; and diaphragmatic, 17 mm. No echocardiographic signs of tamponade (e.g., right atrial or right ventricular diastolic collapse) or constriction were observed. Chamber dimensions were within normal limits except for a mildly enlarged left atrium (LA diameter 39 mm; left ventricular end-diastolic diameter 47 mm; right ventricular diameter 22 mm; right atrial diameter 38 mm). Left ventricular systolic function was preserved, with an ejection fraction (LVEF) of 70% (Figures 1B,C). A computed tomography scan of the chest revealed moderate pericardial effusion (Figure 1D) with a small inflammatory nodule at the left lower lobe (Figure 1E). Pericardiocentesis was performed (Figure 1F). Following the aspiration of 350 mL of chylous pericardial fluid (Figures 1G,H), the procedure was completed with drain placement and absorbable suture closure. Pericardiocentesis was performed under ultrasound guidance, and a 6F pigtail drainage catheter was placed. Per local clinical protocol for chylopericardium, the drain was maintained until output decreased to less than 25–50 mL over 24 h, which was achieved by day 3 post-procedure. Drainage volumes were not systematically recorded as this was not part of the prospective data collection. The pericardial fluid appeared grossly milky and creamy white. Biochemical analysis of the available sample revealed: total protein 56.8 g/L, albumin 39.1 g/L, glucose 5.43 mmol/L, sodium 131.7 mmol/L, chloride 97.8 mmol/L, potassium 3.39 mmol/L, lactate dehydrogenase 113 IU/L, and adenosine deaminase 3.8 IU/L. A chylous qualitative test was positive. The patient declined further diagnostic testing on the pericardial fluid, including triglyceride and cholesterol levels, chylomicron analysis, Sudan staining, cell count with differential, bacterial/fungal/mycobacterial cultures, TB-specific testing, and cytologic examination for malignant cells, citing cost concerns, a strong desire for early discharge, and the perception that additional testing would not alter his clinical management (perceived low yield) after his symptoms had resolved following drainage. Consequently, these data were not obtained. Despite this limitation, the presumptive diagnosis of idiopathic chylopericardium was supported by the classic milky appearance, positive chylous qualitative test, and comprehensive exclusion of secondary causes (infection, malignancy, trauma, autoimmune disease) through clinical evaluation, imaging, and serologic testing. The patient subsequently demonstrated an uncomplicated recovery.

**Figure 1:**
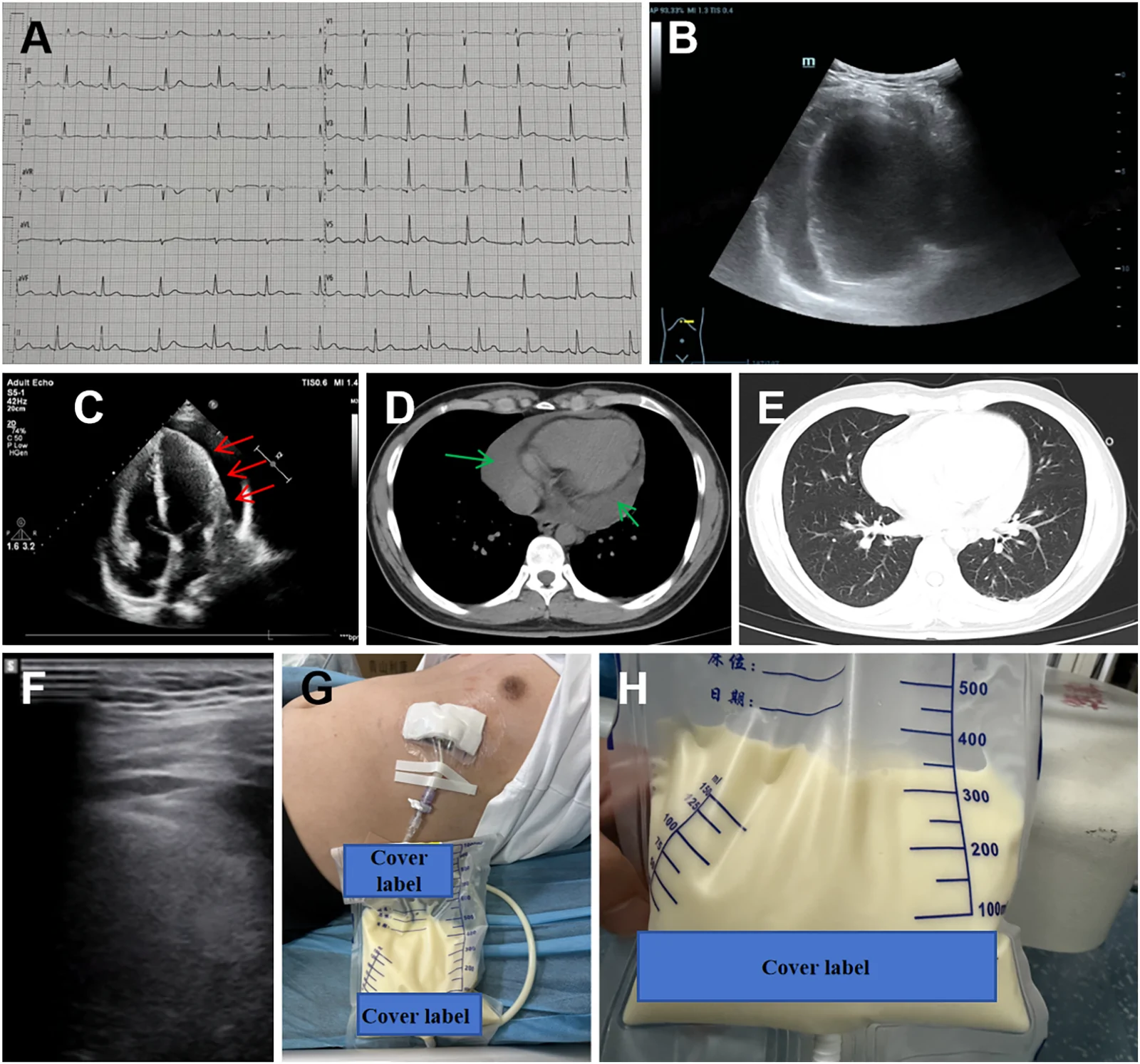
Multimodal imaging showing chylopericardium. **(A)** Electrocardiogram; **(B)** Cardiac ultrasound finding; **(C)** Echocardiographic signs: pericardium effusion (Red arrow); **(D,E)** Chest computed tomography: pericardium effusion (Green arrow); **(F)** Ultrasonography-guided pericardiocentesis; **(G,H)** Milky/creamy white pericardial fluid typical of chylous pericardial effusion. Detailed measurements of the pericardial effusion size are provided in the Supplementary Materials.

Lymphoscintigraphy was performed to evaluate the thoracic duct and identify potential lymphatic sources of the chylous effusion. The study revealed cessation of radiotracer flow at the supradiaphragmatic level, with no tracer accumulation within the pericardial cavity or thoracic region. This pattern indicates functional interruption or delayed transit of lymphatic flow at the supradiaphragmatic level, rather than direct extravasation of chyle into the pericardial space. In the absence of definite pericardial tracer activity, direct lymphatic leakage into the pericardium was not demonstrated by this imaging modality (Figure 2). These findings are most consistent with functional obstruction or incompetence of the thoracic duct at the supradiaphragmatic level.

**Figure 2:**
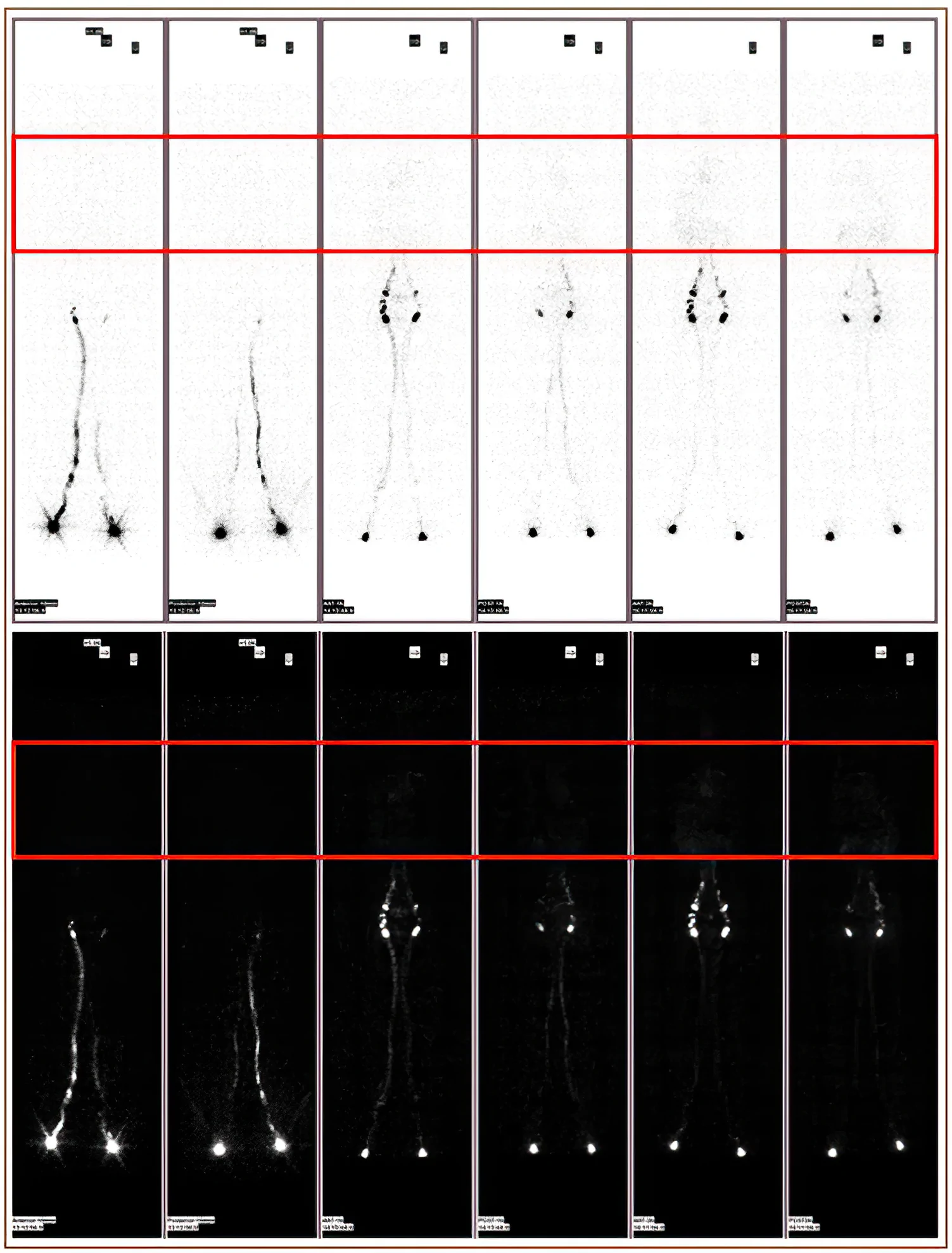
Lymphoscintigraphy. Lymphoscintigraphy demonstrated cessation of radiotracer transit at the supradiaphragmatic level, without definite tracer accumulation within the pericardial cavity or thoracic region (red square frame). This pattern is consistent with functional interruption or delayed lymphatic flow at the supradiaphragmatic level, rather than direct extravasation of chyle into the pericardial space.

The patient's history reveals no prior surgeries, trauma, malignancies, tuberculosis, HIV infection, significant travel. Additionally, the absence of lymphadenopathy and SVC obstruction was identified on imaging. Chest imaging suggests that only the pericardium has fluid accumulation (Supplementary Materials). Subsequently, idiopathic chylopericardium was considered. Patients without cardiac tamponade can initially be treated with dietary modification while patients with cardiac tamponade require urgent fluid removal. The patient was managed conservatively without surgical intervention. Based on published protocols for chylous effusions, treatment consisted of dietary fat restriction combined with octreotide administration (1, 4). A fat-restricted diet supplemented with medium-chain triglycerides (MCT) was initiated, as MCTs are absorbed directly into the portal system rather than via intestinal lymphatics, thereby reducing chyle production (4). Octreotide was administered subcutaneously at a dose of 100 μg three times daily, a regimen previously reported for chylopericardium. The duration of octreotide therapy was approximately two weeks, consistent with the published case. No adverse effects, including glucose dysregulation or gastrointestinal symptoms, were observed during treatment. The patient demonstrated progressive clinical improvement, with complete symptom resolution by the time of hospital discharge. At two-month follow-up, the patient remained completely asymptomatic. Follow-up echocardiography demonstrated complete resolution of pericardial effusion (no fluid visualized in any pericardial space), with normal left ventricular ejection fraction (60%) and normalization of left atrial dimension (LA diameter 32 mm, down from 39 mm at presentation). The patient reported good adherence to dietary modifications, including continued restriction of long-chain fatty acids and use of medium-chain triglyceride supplementation. No adverse effects from octreotide therapy were observed during or after treatment. Comprehensive raw data from the patient's diagnostic evaluation and follow-up are presented in the Supplementary Materials. Together, these supplementary data provide transparent and reproducible documentation supporting the clinical course described herein.

## Discussion

Idiopathic chylopericardium is a rare clinical entity defined by the accumulation of chylous fluid within the pericardial cavity in the absence of an identifiable secondary cause (1, 2). In a systematic review of 33 adult cases reported between 1996 and 2008, idiopathic etiology was the most common, accounting for approximately one-third of all chylopericardium cases (1). A subsequent retrospective series of nine patients with primary idiopathic chylopericardium reported a mean age of 28 years, with dyspnea as the most common presenting symptom (3). Our patient, a 22-year-old male, aligns with this demographic profile. Comprehensive evaluation in our case excluded secondary causes including malignancy, infection, trauma, and autoimmune disease, thereby supporting the idiopathic diagnosis in the absence of a confirmed secondary cause.

The pathophysiology of idiopathic chylopericardium is believed to involve abnormal or damaged lymphatics or elevated pressure within the thoracic duct leading to chyle reflux into the pericardial space (4). Proposed mechanisms include incompetence of lymphatic valves at the thoracic duct–pericardial junction and congenitally abnormal communications (3). The established pathophysiology of idiopathic chylopericardium thus centers on structural or functional lymphatic abnormalities, independent of dietary factors. In our patient, symptom onset occurred approximately one week after he began consuming a self-reported high-fat diet. While this temporal sequence is intriguing, it does not establish causation. Importantly, the following mechanistic proposal is speculative and is not proven by our data; it is intended to generate hypotheses rather than to establish causation. One speculative mechanism, though unproven in this case, is that a sustained increase in dietary fat intake may transiently elevate chyle production, potentially increasing intraluminal pressure within the thoracic duct and, in susceptible individuals with underlying (but radiologically occult) lymphatic abnormalities, could theoretically promote retrograde chylous flow into the pericardial cavity (4). However, several important limitations preclude a causal interpretation: the dietary exposure was not prospectively quantified, the serum triglyceride level at presentation was only modestly elevated (3.17 mmol/L, normal <1.70 mmol/L), and lymphoscintigraphy did not demonstrate direct evidence of pericardial lymphatic leakage or a definitive fistulous tract. Therefore, the current evidence supports only a temporal association, not a causal relationship, between the dietary binge and the development of chylopericardium. This mechanistic hypothesis should be viewed as hypothesis-generating and requires further investigation in future studies. The lymphoscintigraphic finding of supradiaphragmatic flow cessation without frank extravasation supports functional obstruction rather than an overt anatomic fistulous tract.

Management of idiopathic chylopericardium lacks established guidelines and remains controversial (4). Conservative therapy typically includes pericardiocentesis, a medium-chain triglyceride-rich diet, and octreotide administration (2, 4). Octreotide, a somatostatin analogue, reduces splanchnic blood flow and lymphatic output (4). However, the failure rate of conservative management is substantial. Dib et al. reported that 57% of patients (16 of 28) receiving initial conservative therapy ultimately required surgical intervention (1). Similarly, Han et al. found that six of nine patients (67%) failed conservative treatment and required surgery (3). Makarian et al. described an 18-year-old male with persistent idiopathic chylopericardium for five months who failed conservative management and ultimately required thoracoscopic thoracic duct ligation and pericardial window creation, which subsequently revealed an underlying generalized lymphatic anomaly (5). More recently, retrograde thoracic duct embolization has emerged as a minimally invasive alternative for refractory cases (6). Our patient responded favorably to conservative therapy, which we attribute to the acute and reversible nature of the inciting dietary trigger rather than a chronic anatomic abnormality.

Despite the successful outcome in this case, caution is warranted. Cardiac tamponade can occur as a presenting manifestation of idiopathic chylopericardium (7), and recurrence after initial successful treatment has been documented (8). Our patient's two-month recurrence-free interval is encouraging but insufficient to rule out late recurrence. Close clinical follow-up with serial echocardiography is therefore essential.

A central requirement for diagnosing chylopericardium is complete pericardial fluid analysis, including triglyceride and cholesterol levels, chylomicrons or Sudan staining, cell count with differential, cultures, TB testing, and cytology (1, 5). In the present case, these tests were not performed because the patient declined them after symptom resolution, citing financial constraints, a strong desire for early discharge, and the perception that additional results would not alter his treatment plan. It is a not uncommon scenario in real-world clinical practice.

The differential diagnosis of chylopericardium includes cholesterol pericarditis and purulent pericardial effusion due to infection such as tuberculosis. Fluid analysis in cholesterol pericarditis will show cholesterol crystals and a lower concentration of triglyceride (9, 10). Cholesterol pericarditis, a rare condition often associated with long-standing hypothyroidism or rheumatoid arthritis, is characterized by a ‘gold paint’ effusion with cholesterol crystals and markedly elevated cholesterol levels (>100 mg/dL) but normal or low triglyceride levels, distinguishing it from chylopericardium (9). Meanwhile, pericardial effusion due to infection, such as tuberculous pericarditis, typically shows higher neutrophils than lymphocytes in cellular content (especially in early stages), positive culture for the organism, and lower triglyceride levels (3, 7). Tuberculous pericarditis, the most common cause of large pericardial effusion in tuberculosis-endemic regions, usually presents with fever, night sweats, and elevated adenosine deaminase (ADA) levels, with definitive diagnosis requiring culture or PCR for Mycobacterium tuberculosis (3, 7). In our patient, the absence of a prior tuberculosis diagnosis or any typical clinical manifestations of tuberculosis (e.g., fever, night sweats, weight loss), together with a normal ADA level (3.8 IU/L, well below the typical cutoff of >40 IU/L for tuberculous pericarditis), and the milky-white appearance of the fluid, effectively ruled out these alternative diagnoses (7).

A stepwise diagnostic approach for idiopathic chylopericardium is proposed based on our experience and the literature. First, pericardial effusion should be confirmed by echocardiography or chest CT. Second, diagnostic pericardiocentesis should be performed; the fluid appearance (milky/creamy white) and a positive chylous qualitative test or elevated triglyceride level (>1.24 mmol/L) support chylopericardium (1, 8). Third, secondary causes—including malignancy, infection (tuberculosis, fungal, viral), autoimmune disease, trauma, and prior cardiothoracic surgery—must be systematically excluded through clinical history, serologic testing (ANA, HIV, EBV, IGRA/TB-PCR), and imaging (CT chest to rule out mediastinal mass or lymphadenopathy) (4, 5). Fourth, lymphoscintigraphy or non-contrast MR lymphangiography can be considered to identify lymphatic leakage or obstruction (11). Finally, if all evaluations are negative, idiopathic chylopericardium can be presumptively diagnosed (4). While complete fluid biochemical analysis is ideal, in consent-limited or resource-constrained settings, a pragmatic diagnosis based on macroscopic appearance, exclusion of secondary causes, and response to conservative therapy remains acceptable, as demonstrated in this case.

In the present case, complete fluid analysis was not performed due to patient refusal. However, using the diagnostic framework summarized in Table 1 (12–17), Although complete biochemical confirmation was not available, the available data: milky-white appearance, positive chylous qualitative test, normal ADA (3.8 IU/L, well below the >40 IU/L cutoff for tuberculous pericarditis), negative IGRA, absence of fever or constitutional symptoms, and no mass or lymphadenopathy on imaging, are most consistent with idiopathic chylopericardium. Cholesterol pericarditis is ruled out by the absence of cholesterol crystals and normal cholesterol level; tuberculous pericarditis is ruled out by normal ADA, negative IGRA, and lack of clinical symptoms; and malignant effusion is ruled out by negative imaging findings. According to the Dib diagnostic scoring system (milky fluid, lymphocytic predominance, negative culture and cholesterol/triglyceride <1), our case would achieve a diagnostic score of at least 2 (1, 2). Thus, despite the absence of gold-standard triglyceride measurement, the presumptive diagnosis remains reasonable and clinically supportable under the circumstances.

**Table 1:** Differential diagnosis of pericardial effusion based on fluid analysis, imaging, and diagnostic criteria.

Fluid feature	Idiopathic chylopericardium	Cholesterol pericarditis	Tuberculous pericarditis	Malignant pericardial effusion
Gross appearance	Milky white, creamy; may be pinkish or bloody after centrifugation (1, 12)	Gold paint/yellow-brown, turbid (9, 13)	Serous/serosanguinous (14)	Serosanguinous/hemorrhagic (1)
Triglyceride level	> 500 mg/dL (>5.65 mmol/L) (1, 2)	Low/normal (9, 13)	Low/normal	Low/normal (1)
Cholesterol level	Normal or low; cholesterol/triglyceride ratio < 1 (1)	> 70–100 mg/dL, cholesterol crystals present (9, 13)	Normal	Variable
Cell count/ differential	Lymphocyte predominance (>70–80%) (1, 12)	Variable; foam cells, cholesterol clefts (9, 13)	Lymphocytes (chronic phase)	Often lymphocytic or malignant cells (1)
ADA level	Normal (< 10 IU/L) (12)	Normal (9, 13)	Elevated (> 40 IU/L) but may be normal in early stages (14, 15)	Variable (can be elevated, not specific)
Cytology	Negative for malignant cells (1)	Negative; cholesterol crystals diagnostic (9)	Negative (granulomas in tissue)	Positive in ∼70–80% (1)
Gram stain/Culture	Negative (1)	Negative	Positive for M. tuberculosis in 30–50%	Negative unless superinfected
Lymphoscintigraphy/CTL	Abnormal radiotracer distribution; classified into 3 types based on lymphatic drainage patterns (10, 16)	Not indicated	Not indicated	May show mass effect
Diagnostic criteria	Dib score ≥2: milky fluid, TG > 500 mg/dL, cholesterol/TG < 1, lymphocytic predominance, negative culture (1)	Cholesterol crystals + cholesterol >70 mg/dL + exclusion of other causes (9, 13)	Culture/PCR positive; or clinical criteria with elevated ADA	Cytology positive; or known malignancy + typical findings (1)
Clinical context	No secondary cause; often young adults (1, 9, 12)	Longstanding hypothyroidism, rheumatoid arthritis, hyperlipidemia	Fever, night sweats, weight loss, endemic area, HIV (17)	Known malignancy, lymphadenopathy, older age (1)

Interestingly, the lymphoscintigraphic finding of supradiaphragmatic flow cessation without frank pericardial tracer accumulation supports a mechanism of functional obstruction or delayed lymphatic transit, rather than an overt anatomic fistulous tract. This pattern interrupted flow without direct extravasation that has been described in idiopathic chylopericardium and may reflect elevated intraluminal pressure within the thoracic duct or incompetence of lymphatic valves, leading to retrograde chylous flow into the pericardium via small mediastinal lymphatic collaterals that may not be detectable by lymphoscintigraphy (4, 16).

However, several treatment parameters, including daily drain output, total caloric intake, and the precise response timeline, were not prospectively recorded in this retrospective case report. Future prospective studies should include standardized documentation of these variables to better characterize treatment response in idiopathic chylopericardium.

The two-month follow-up period in this case, while demonstrating initial treatment success, is insufficient given the natural history of idiopathic chylopericardium. A systematic review by Verma et al. encompassing 98 patients reported a median follow-up duration of 180 days (IQR 180–377 days), with chylopericardium recurrence occurring in 16% of cases and 10% of patients requiring rehospitalization (2). This 16% recurrence rate underscores the importance of extended surveillance beyond the initial post-treatment period.

Contemporary evidence on chylopericardium remains limited to case reports and small series. Recent advances in cardiac lymphatic research have established that lymphatic dysfunction is emerging as a hallmark of cardiovascular disease, contributing to insufficient clearance of interstitial fluid and immune cells from the heart (18). This framework supports the concept: underlying but radiologically occult lymphatic abnormalities that may predispose individuals to chylopericardium when challenged by increased chyle production. Regarding management outcomes, a systematic review of 45 patients reported that pericardiocentesis alone is frequently followed by recurrence or need for further intervention (2), and a recent evidence-based review emphasized that conservative therapy can be effective in appropriately selected patients, though a multidisciplinary strategy is critical to guide escalation to surgical intervention when indicated (19). Recent study further proposed quantitative thresholds for surgical intervention including pericardial drainage exceeding 1,500 mL per day, persisting above 500 mL per day after five days, or leading to malnutrition (2). The role of lymphoscintigraphy in diagnosing idiopathic chylopericardium has also been described, with findings of supradiaphragmatic flow cessation without frank extravasation supporting functional obstruction rather than an overt anatomic fistulous tract (20). A systematic review of traumatic chylothorax reported that high-output cases (>1,000 mL/day) often required surgical intervention after an average of 12.5 days of conservative management, underscoring the importance of clear escalation thresholds and multidisciplinary coordination (21). The 2024 ACR Appropriateness Criteria further endorse a multidisciplinary, etiology-based approach to treatment planning, with lymphangiography and MR lymphangiography recommended as appropriate diagnostic tools (22). Taken together, these contemporary data reinforce that while conservative management remains a reasonable initial strategy in hemodynamically stable patients, prompt surgical referral should be considered when clinical response is inadequate, and long-term follow-up is essential given the reported recurrence rates (2).

In conclusion, this case highlights a temporal association between extreme short-term high-fat dietary intake and the development of acute idiopathic chylopericardium in a young adult without other identifiable risk factors. Whether dietary factors can serve as a precipitating trigger remains speculative and requires further investigation. Conservative management with dietary fat restriction and octreotide is a reasonable initial strategy in hemodynamically stable patients. However, given the reported failure rate of conservative therapy, surgical intervention with thoracic duct ligation and pericardial window creation should be considered promptly if symptoms persist or recur (1, 2).

## Conclusion

This case suggests a temporal association between an extreme, short-term high-fat dietary intake and the development of idiopathic chylopericardium in a young adult without identifiable structural lymphatic abnormalities. However, causality cannot be inferred from a single case, and the dietary exposure was not quantified, serum triglyceride elevation was modest, and lymphoscintigraphy did not show a definitive pericardial lymphatic leak. Further studies are needed to explore whether dietary factors may act as a trigger in predisposed individuals. In hemodynamically stable patients without cardiac tamponade, conservative management with pericardiocentesis, a fat-restricted diet, and octreotide can contribute to complete symptom resolution in appropriately selected cases. Given the substantial failure rates of conservative therapy reported in the literature, prompt reevaluation and consideration of surgical intervention remain essential for persistent or recurrent effusion. Long-term clinical follow-up is warranted to monitor for late recurrence.

## Data Availability

The original contributions presented in the study are included in the article/Supplementary Material, further inquiries can be directed to the corresponding author.
