# Supplementary material for: Idiopathic chylopericardium temporally associated with short-term high-fat dietary excess in a young man: a case report
Source: Front Cardiovasc Med. 2026 Oct 8;13:1863669. doi: 10.3389/fcvm.2026.1863669 (PMC13687049; doi:10.3389/fcvm.2026.1863669)

1.Echocardiographic Report

Indication: Discovery and evaluation for idiopathic chylopericardium

Two-Dimensional and M-Mode Measurements (dimensions in mm, area in cm²):

Parameter:
LV (left ventricle) 47
LA (left atrium) 39
RV (right ventricle) 22
RA (right atrium) 38
IVS (interventricular septum) 8
LVPW (left ventricular posterior wall) 8
AO (aorta) 23
AAO (ascending aorta) 22
MPA (main pulmonary artery) 21

Doppler Measurements (velocity in m/s, pressure gradient in mmHg):

Parameter:
E wave 1.2
A wave 0.6
AV (aortic valve) 1.4
PV (pulmonary valve) 0.9

Left Ventricular Function:

Parameter:
EDD (end-diastolic diameter) 47 mm
ESD (end-systolic diameter) 28 mm
EDV (end-diastolic volume) 102 ml
ESV (end-systolic volume) 31 ml
SV (stroke volume) 71 ml
EF (ejection fraction) 70%
FS (fractional shortening) 40%

Findings:
The left atrium was mildly enlarged. The remaining cardiac chambers were normal in size. The aorta and pulmonary artery were within normal diameters. Interventricular septum and left ventricular posterior wall thickness and motion amplitude were normal. At rest, global left ventricular wall motion was mildly incoordinate. No significant abnormalities of the valvular structures were observed. The interatrial and interventricular septa were intact. A moderate-to-large pericardial effusion was present, with the following measurements: posterior pericardial space, 23 mm; apical, 16 mm; left lateral, 21 mm; right lateral, 11 mm; diaphragmatic,

17 mm. The proximal aortic arch and descending aorta showed no significant abnormalities.

Doppler Findings:

Trace tricuspid regurgitation was detected, with a maximum velocity (Vmax) of 2.5 m/s, corresponding to a peak pressure gradient of 26 mmHg. No definite intracardiac shunting was observed.

1. Mildly enlarged left atrium
2. Normal left ventricular systolic function (LVEF 70%)
3. Moderate-to-large pericardial effusion

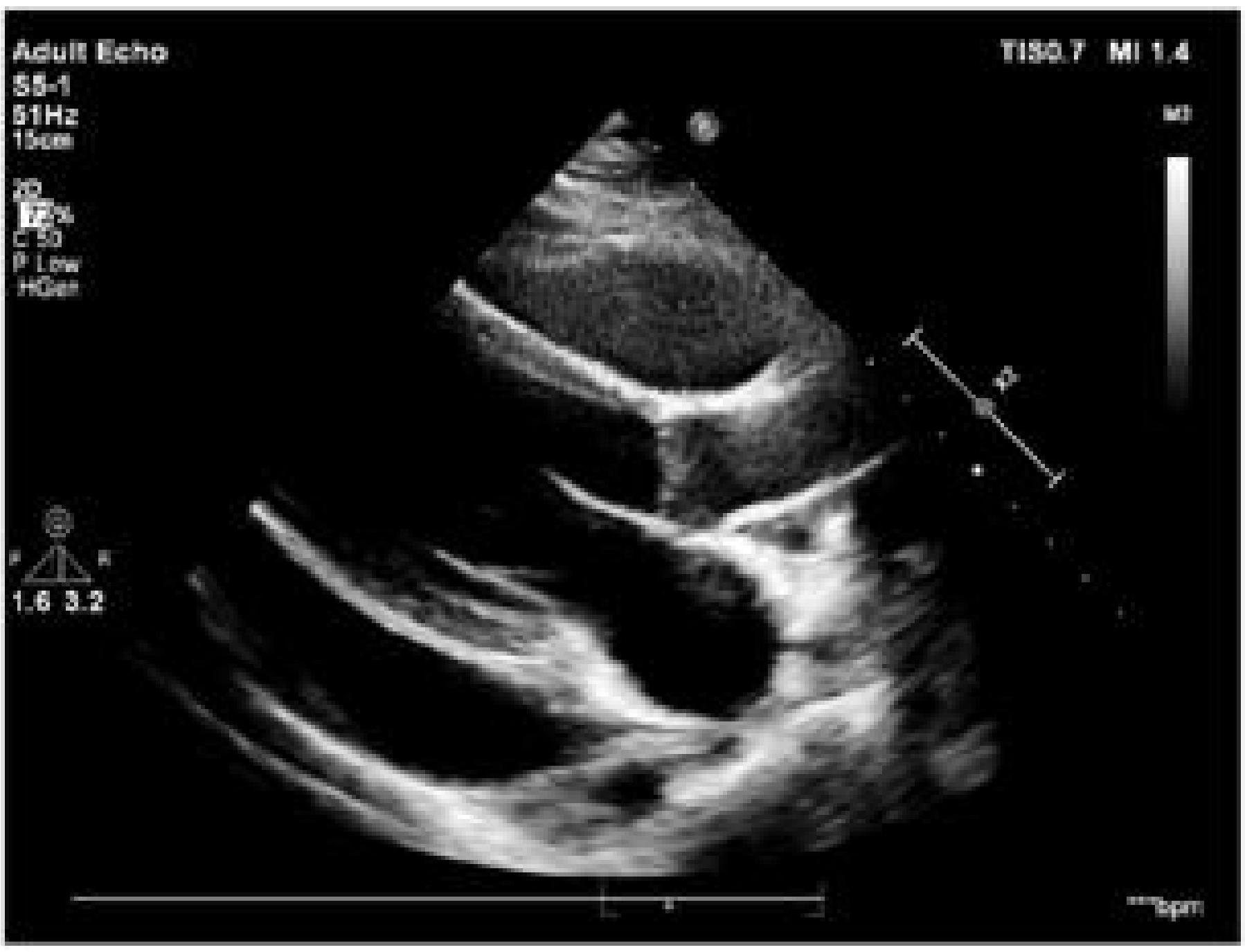

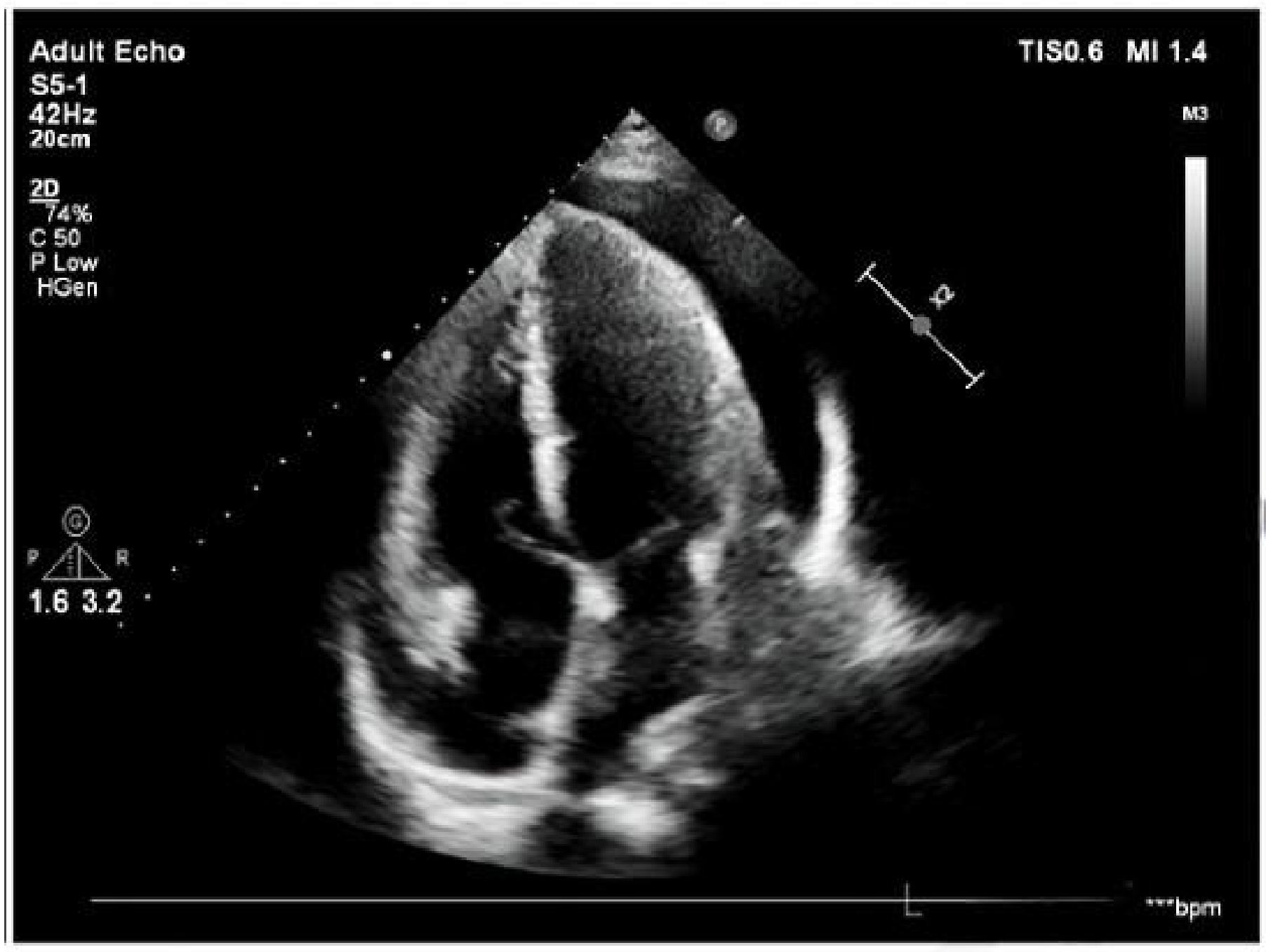

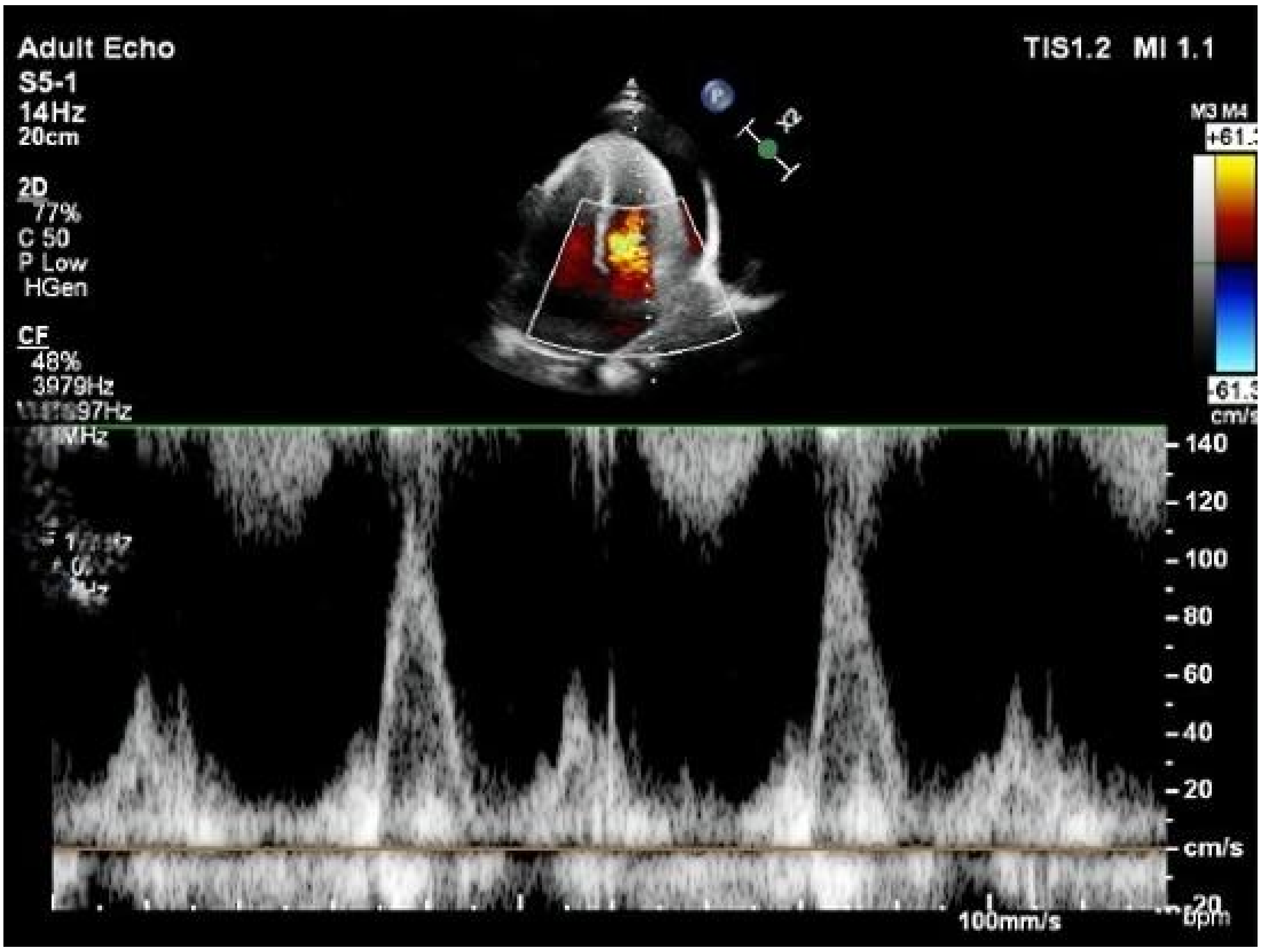

2.Ultrasound-Guided Pericardiocentesis Record

Pre-procedural Ultrasound Examination:

Precordial scanning was performed in the supine position. The pericardial cavity revealed anechoic spaces with the following measurements at end-diastole: apical region, approximately 0.6 cm in depth; posterior pericardial space, approximately 2.0 cm; anterior pericardial space, thin and patchy; lateral pericardial space, approximately 1.9 cm.

Procedure Description:

The patient was placed in the supine position. The puncture area was sterilized and draped with sterile towels. The ultrasound probe was covered with a sterile protective sheath. Local anesthesia was administered using 2% lidocaine. A needle was introduced through the chest wall, and the puncture angle and depth were guided by real-time ultrasound imaging. After accessing the pericardial cavity, milky-white viscous fluid was aspirated. A guidewire was then inserted, followed by sequential dilation of the tract along the guidewire. A 6F drainage catheter was advanced over the guidewire into the pericardial cavity, leaving approximately 4 cm of the catheter external to the skin. The catheter was secured in place and connected to a drainage bag. The patient's vital signs were closely monitored throughout the procedure.

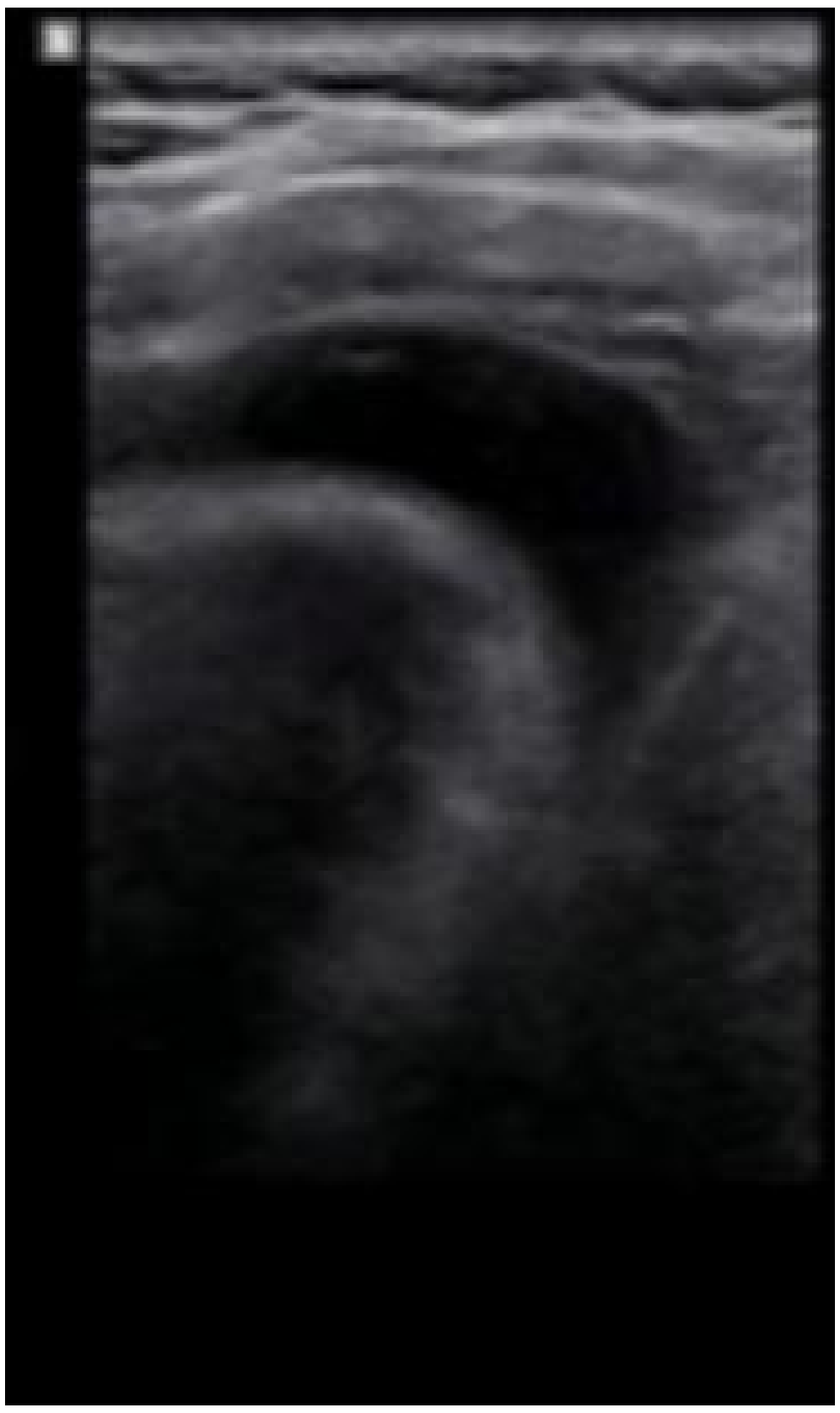

Pericardial cavity (before puncture)

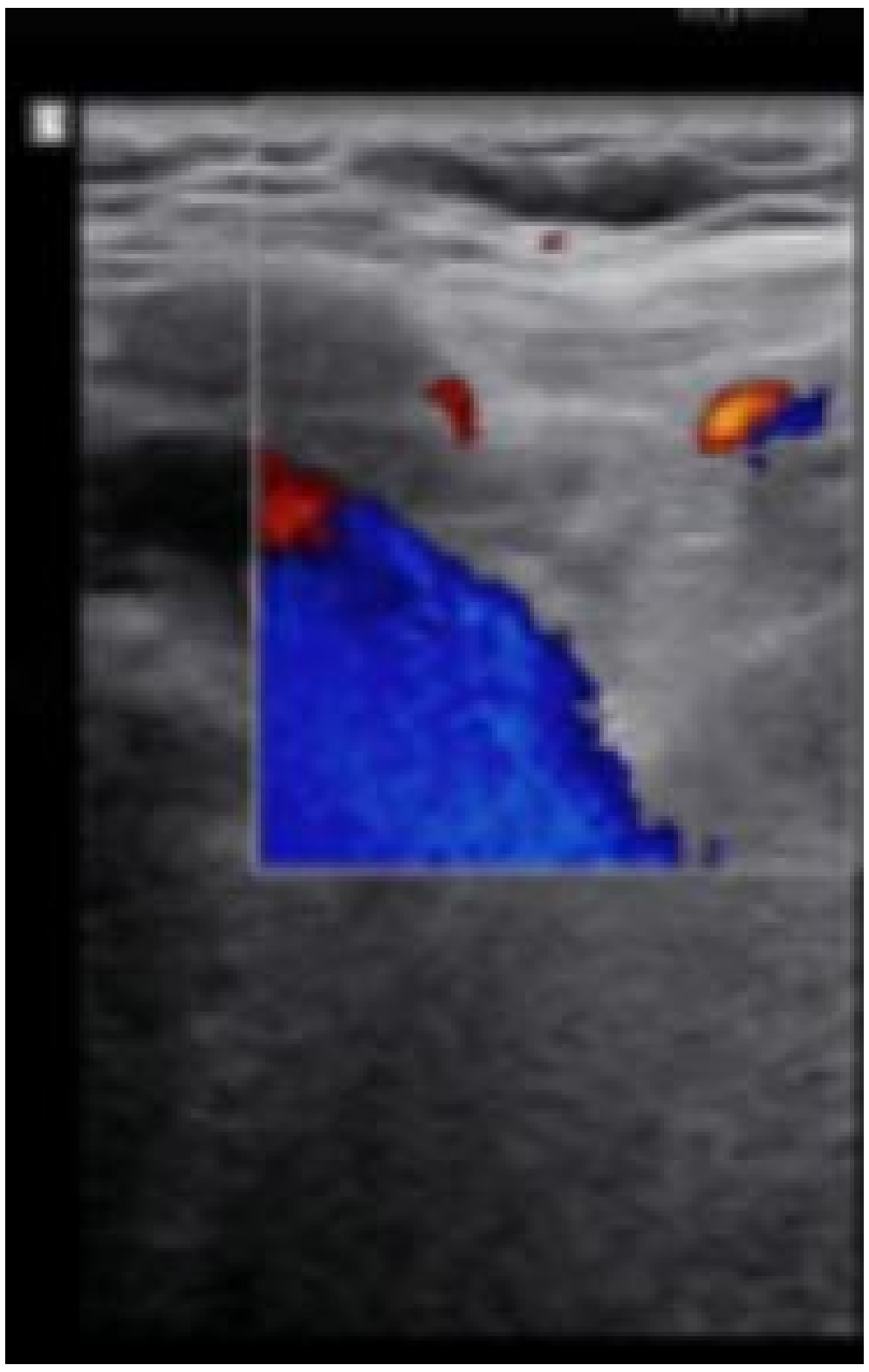

Pericardial cavity (before puncture)

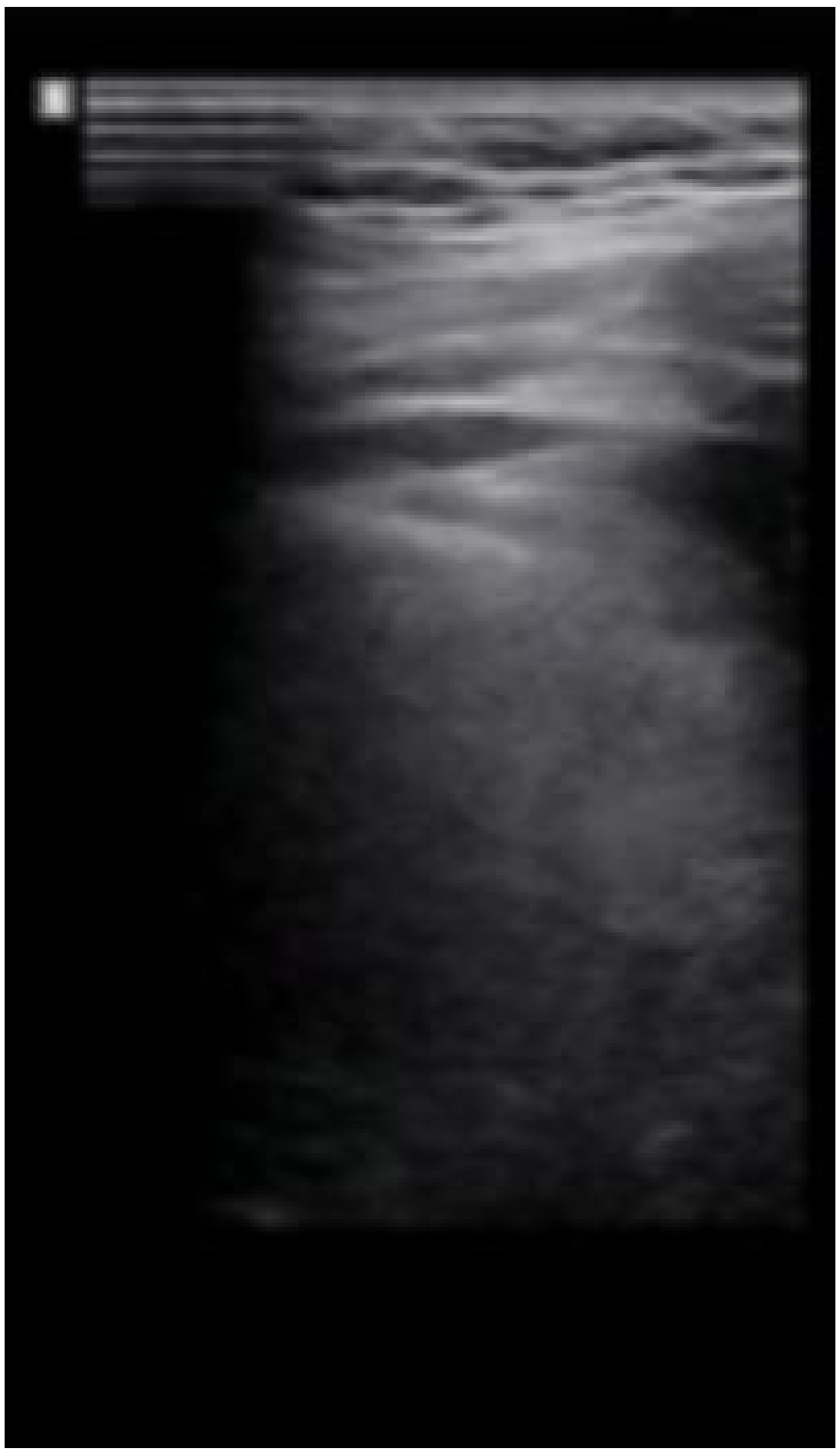

Pericardial cavity (in puncture)

3.Pre-procedural Ultrasound Examination:

Precordial scanning was performed in the supine position. The pericardial cavity revealed anechoic spaces with the following measurements at end-diastole: apical region, approximately 0.6 cm in depth; posterior pericardial space, approximately 2.0 cm; anterior pericardial space, thin and patchy; lateral pericardial space, approximately 1.9 cm.

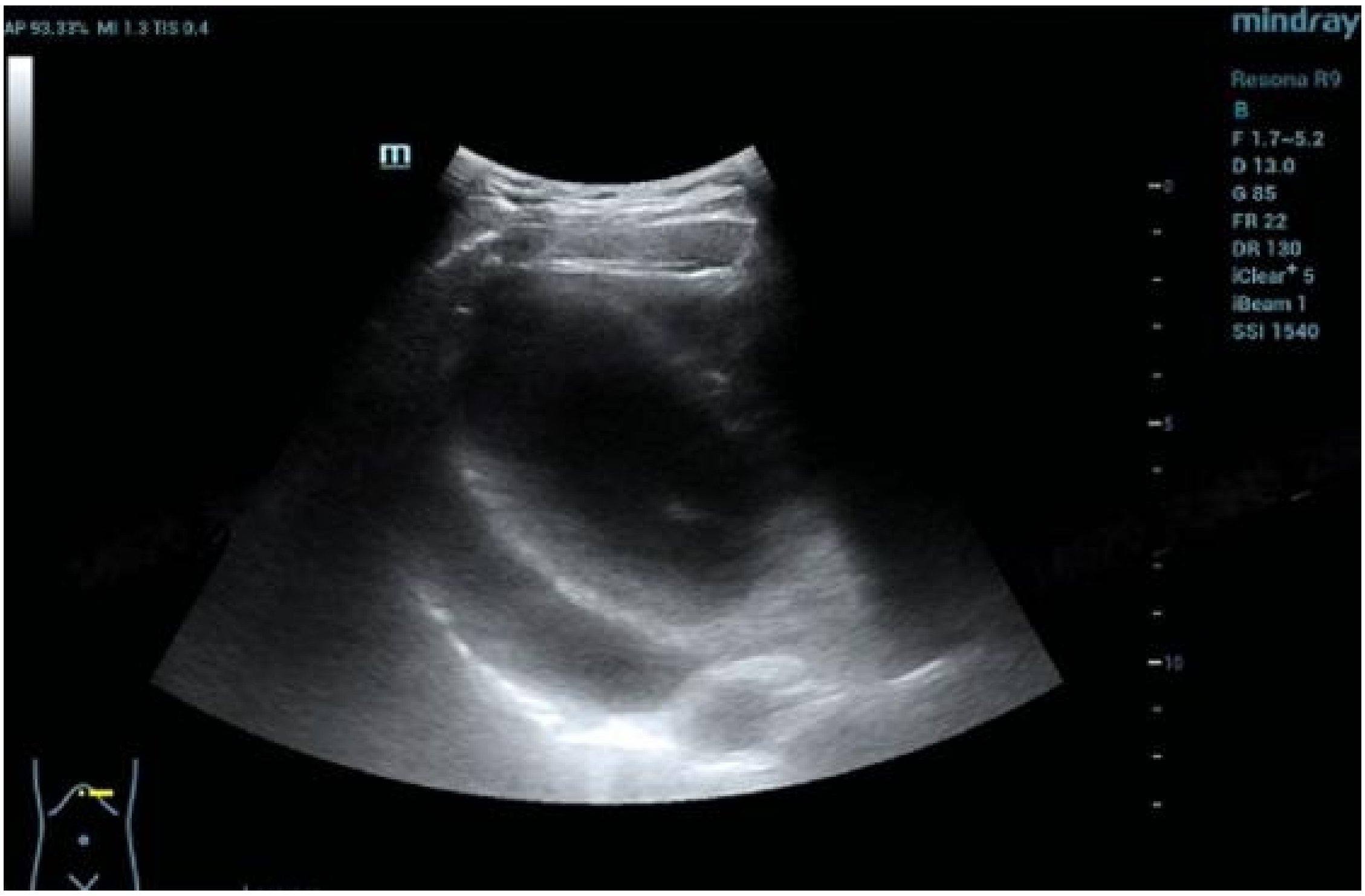

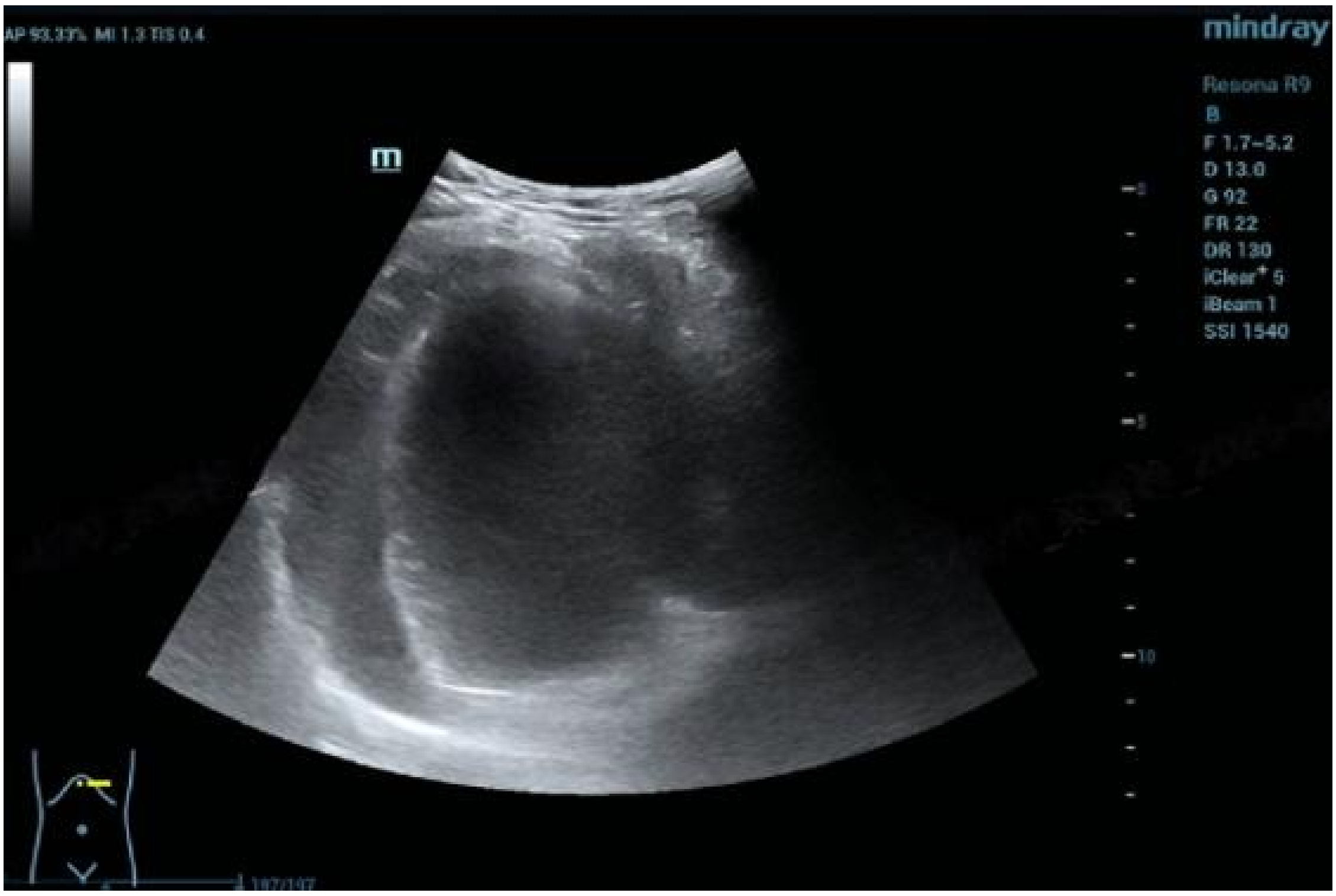

4.Puncture and drainages

Pericardiocentesis was performed under ultrasound guidance, and a 6F pigtail drainage catheter was placed. Per local clinical protocol for chylopericardium, the drain was maintained until output decreased to less than 25-50 mL over 24 hours, which was achieved by day 3 post-procedure.

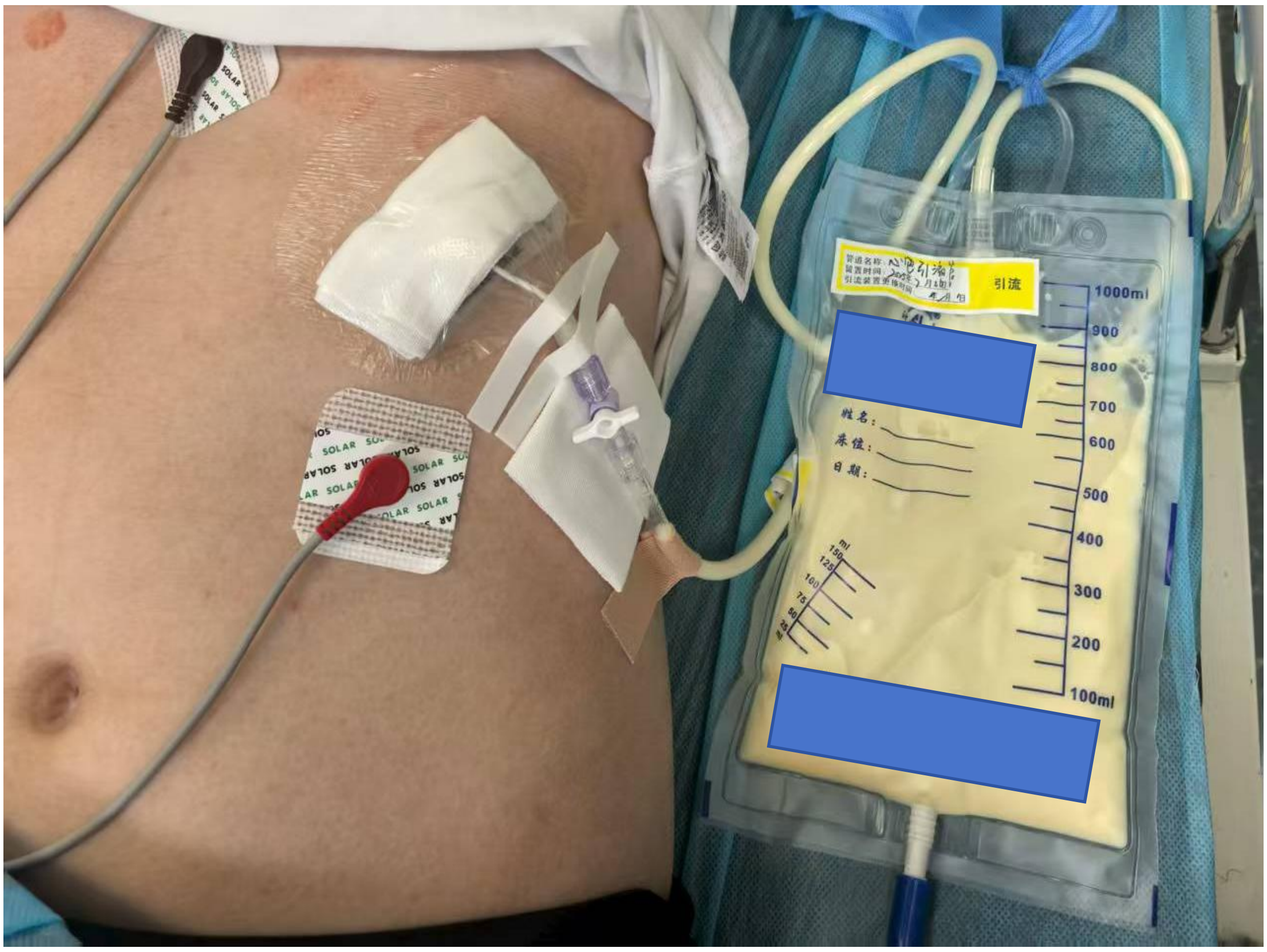

5.Follow-up Echocardiographic Report at 2-month

Indication: Follow-up evaluation for idiopathic chylopericardium

Two-Dimensional and M-Mode Measurements (dimensions in mm, area in cm²):

Parameter:

LV (left ventricle) 44

LA (left atrium) 32

IVS (interventricular septum) 8

LVPW (left ventricular posterior wall) 8

Doppler Measurements (velocity in m/s, pressure gradient in mmHg):

Parameter:

E wave 0.6

A wave 0.5

AV (aortic valve) 1.1

Left Ventricular Function:

Parameter
EF (ejection fraction) 60%

Findings:
All cardiac chambers were normal in size. Interventricular septum and left ventricular posterior wall thickness and motion amplitude were normal. At rest, no definite segmental wall motion abnormalities of the left ventricle were observed. No significant abnormal flow signals were detected across any of the valvular orifices. The pericardial cavity showed no evidence of pericardial effusion. Left ventricular systolic function was normal.

1. Normal cardiac chamber sizes
2. Normal left ventricular systolic function (LVEF 60%)
3. No pericardial effusion

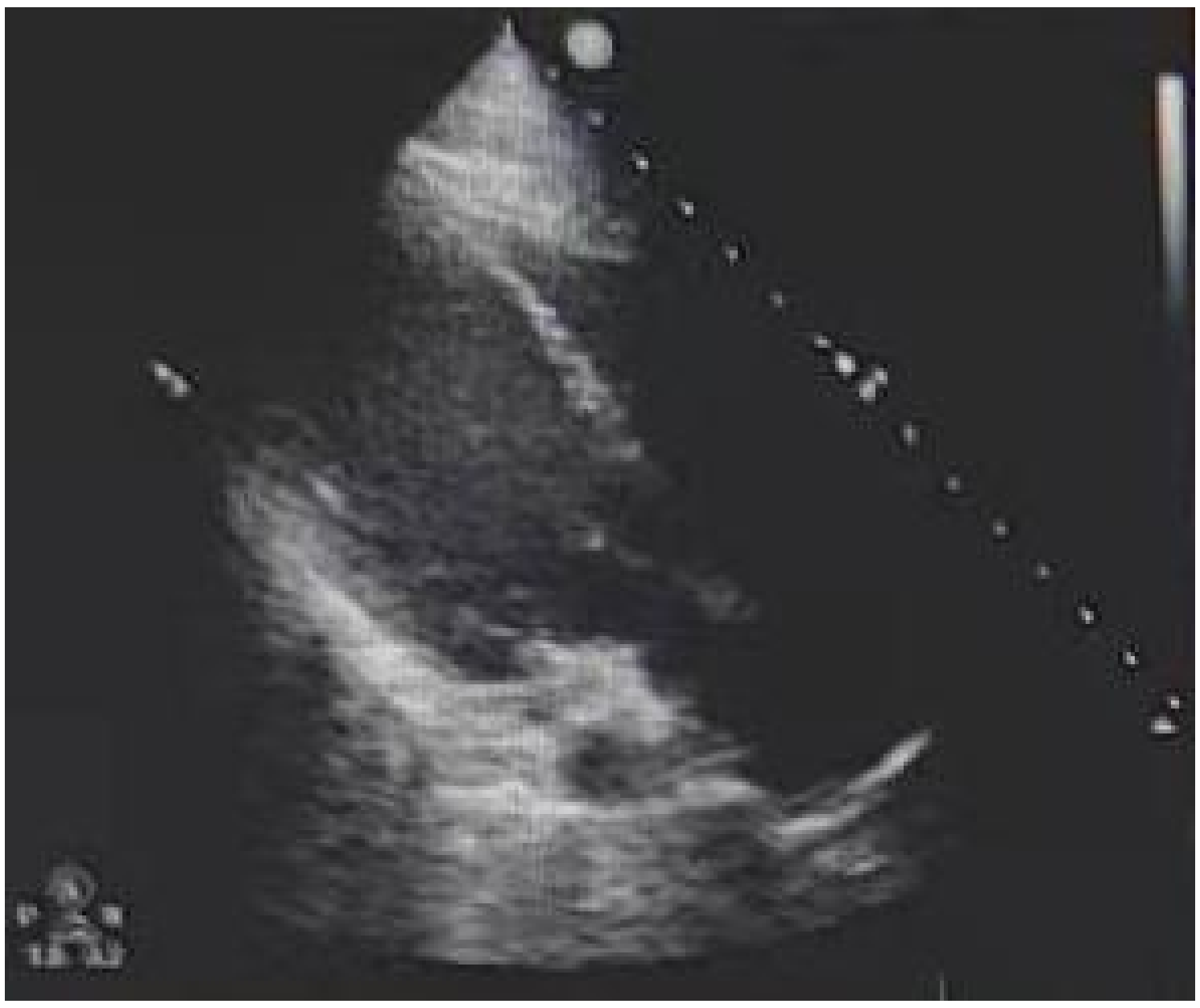

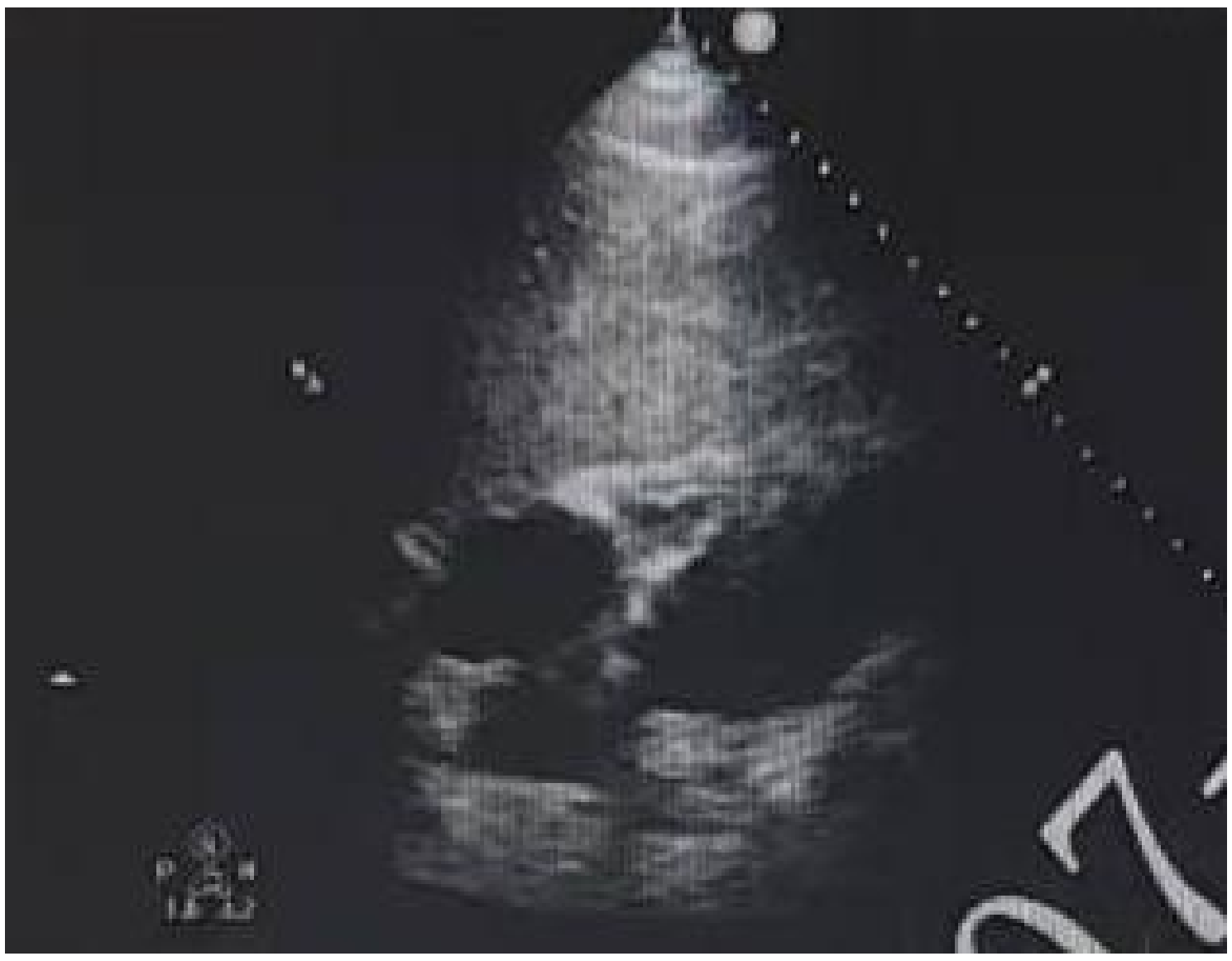

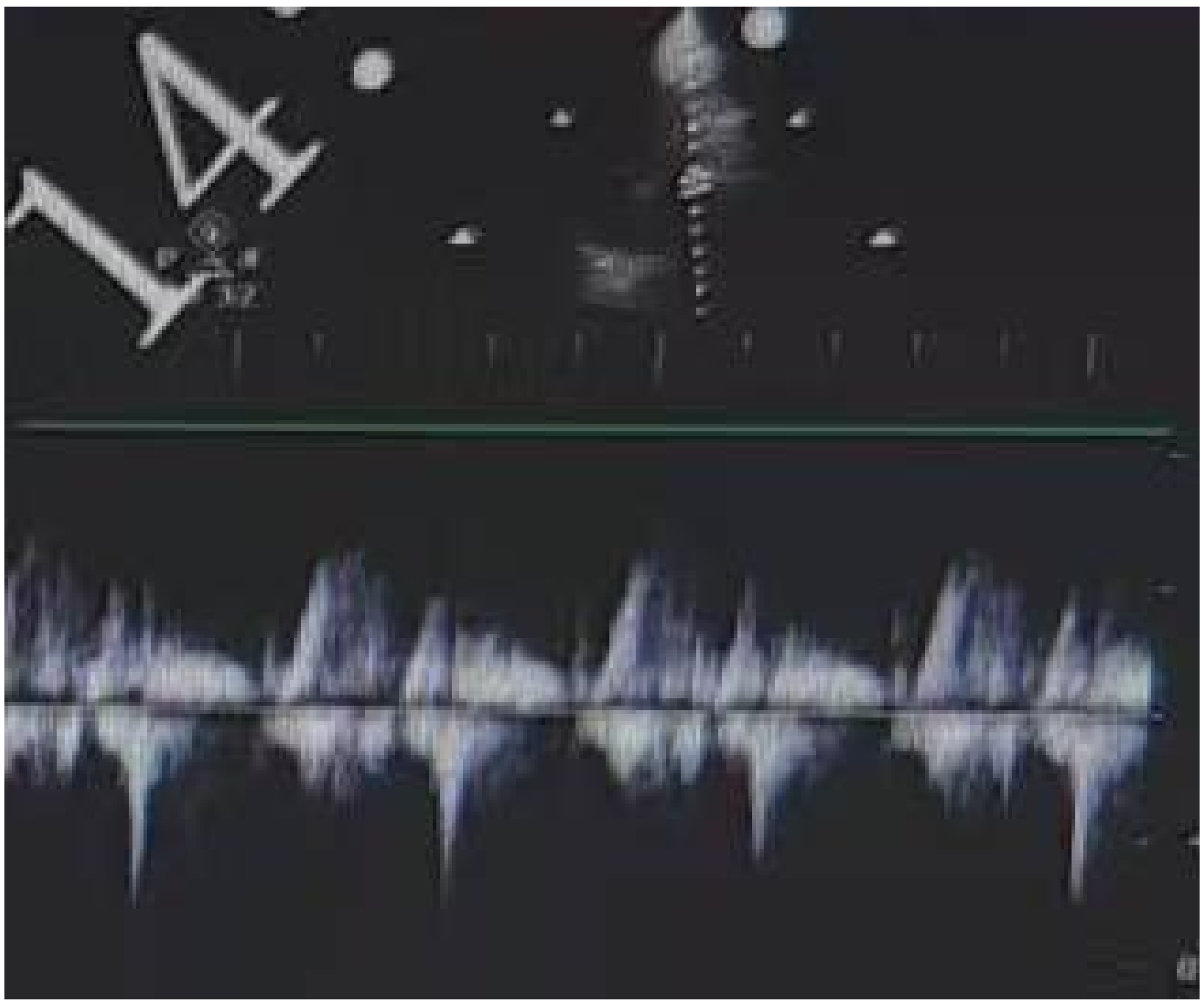

Chest imaging at admission

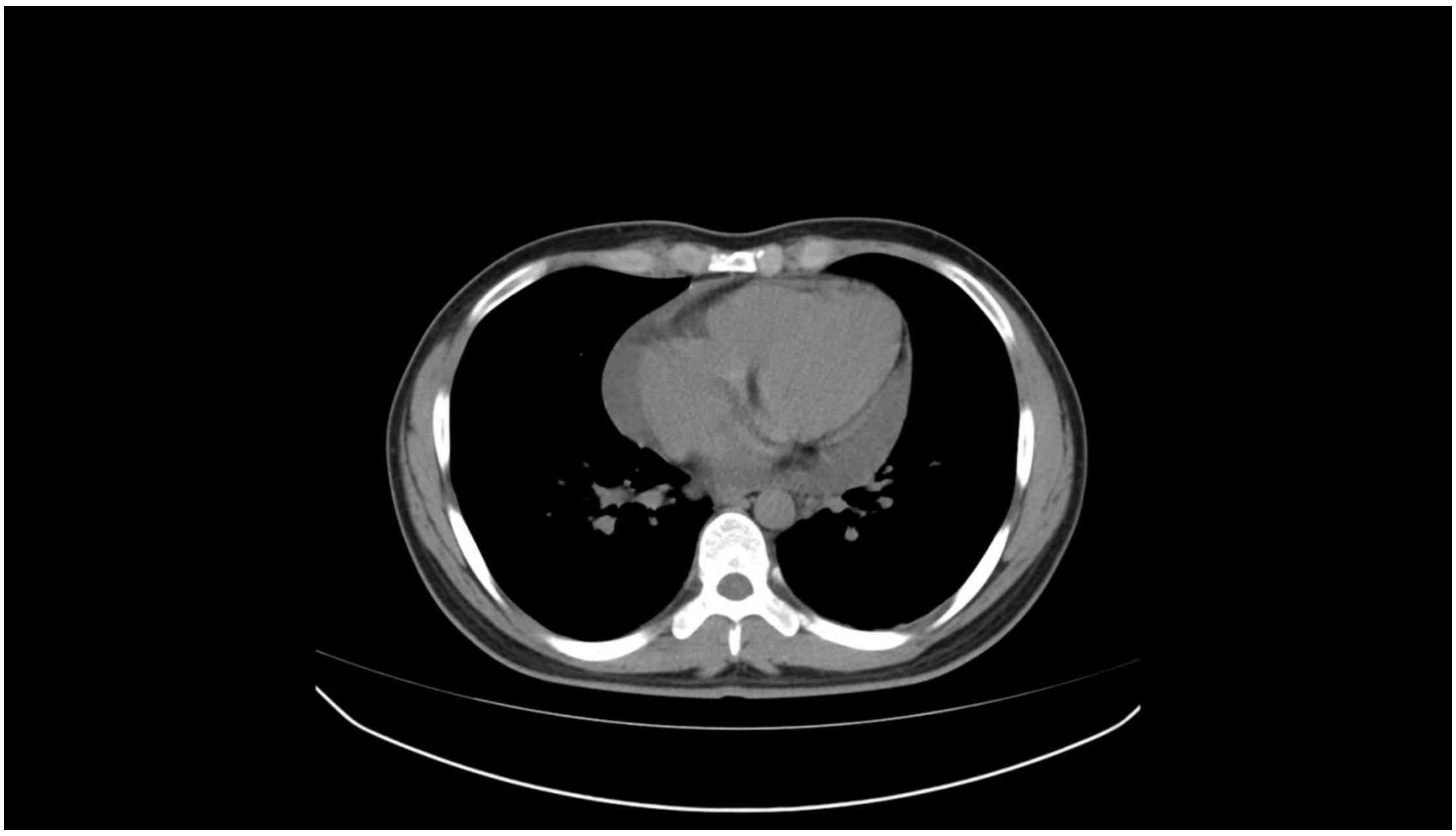

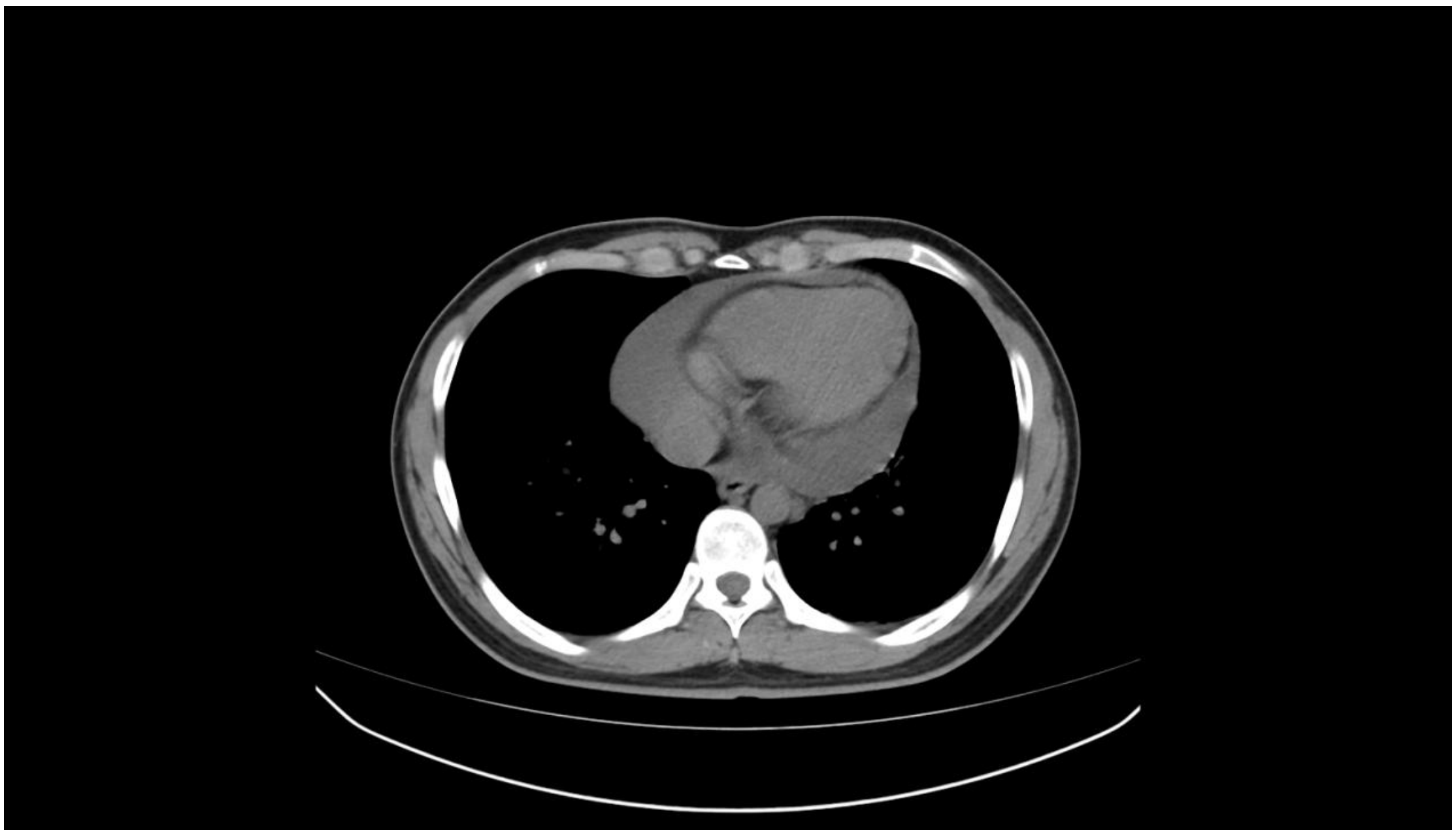
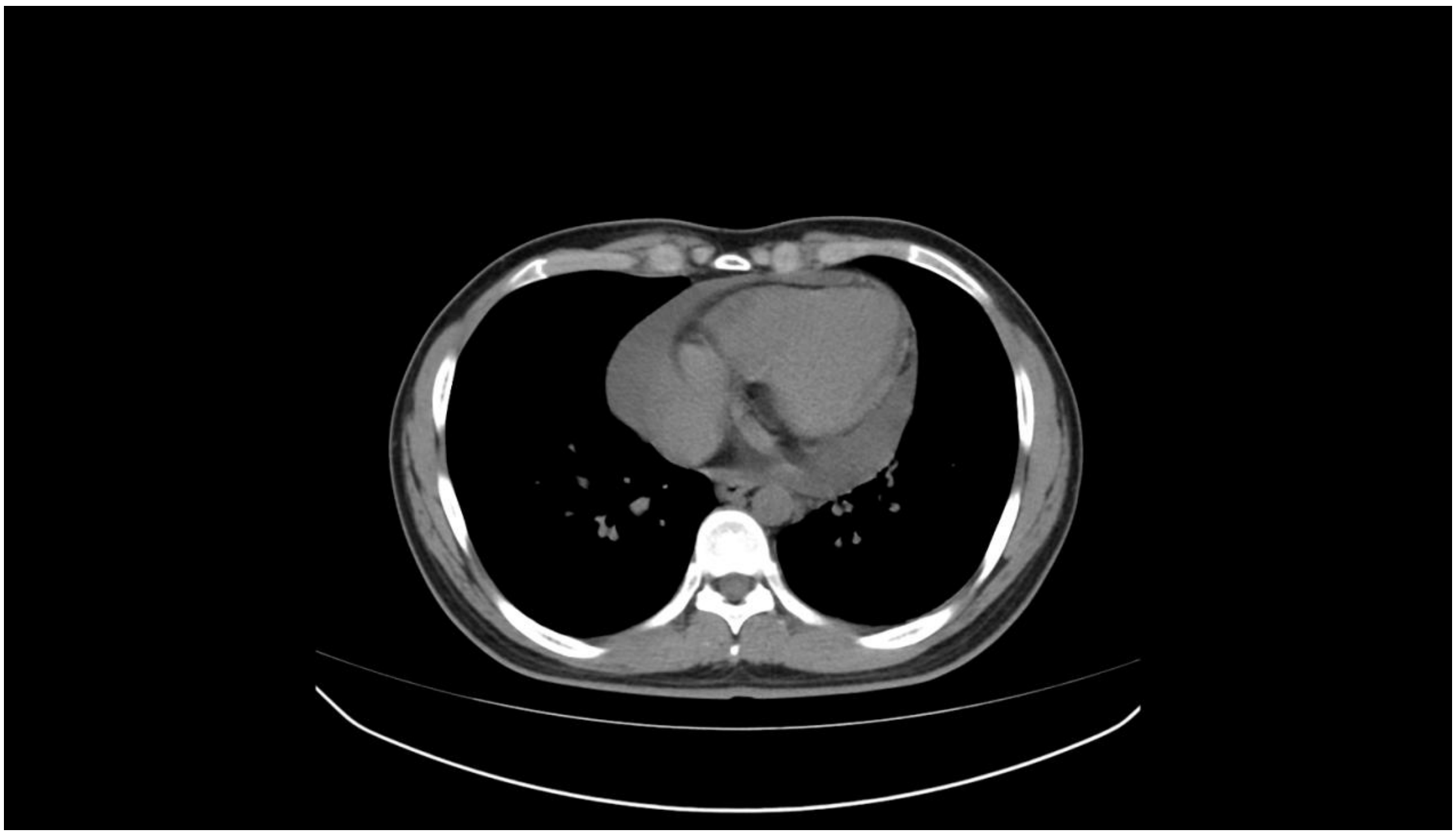

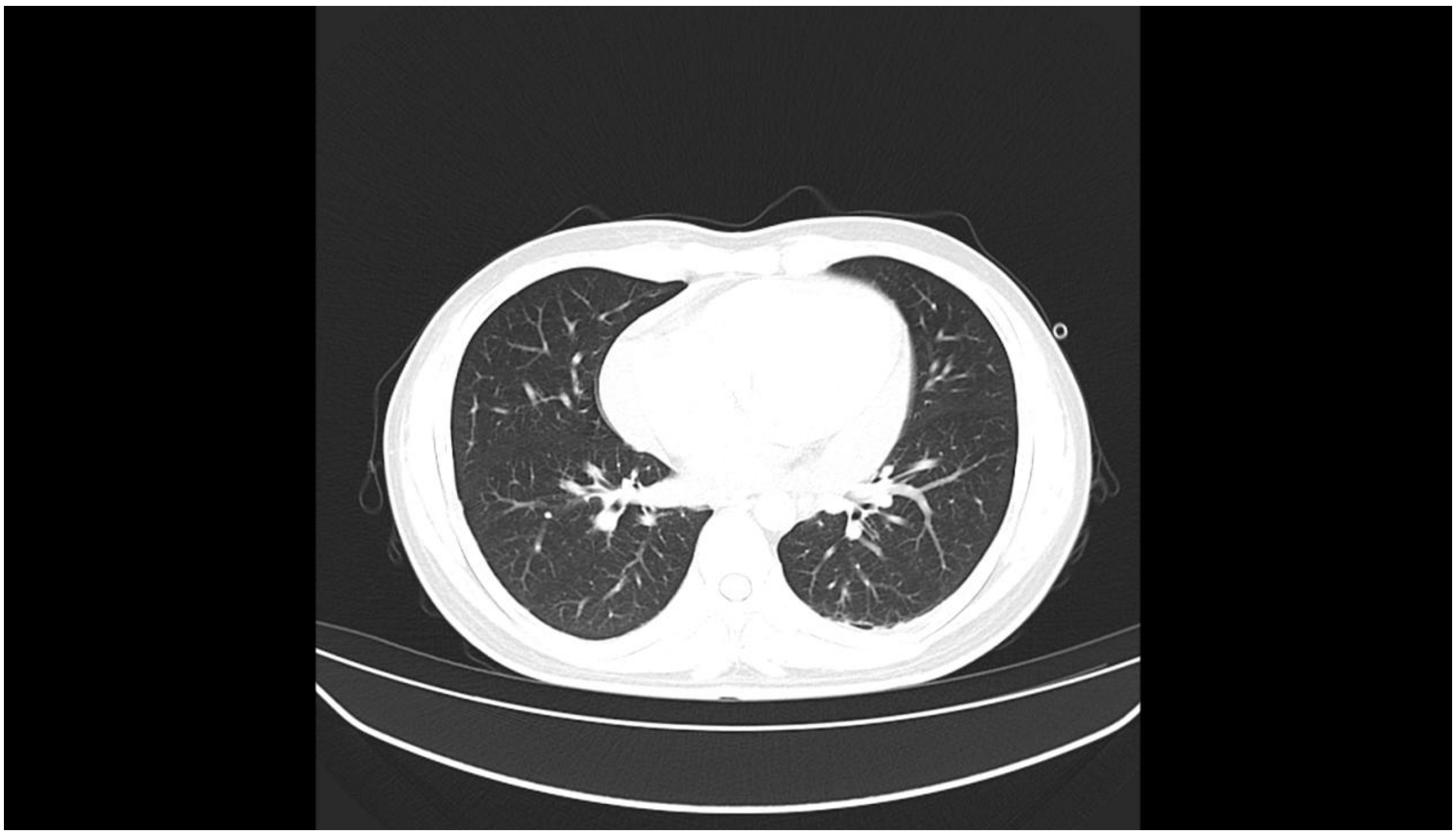

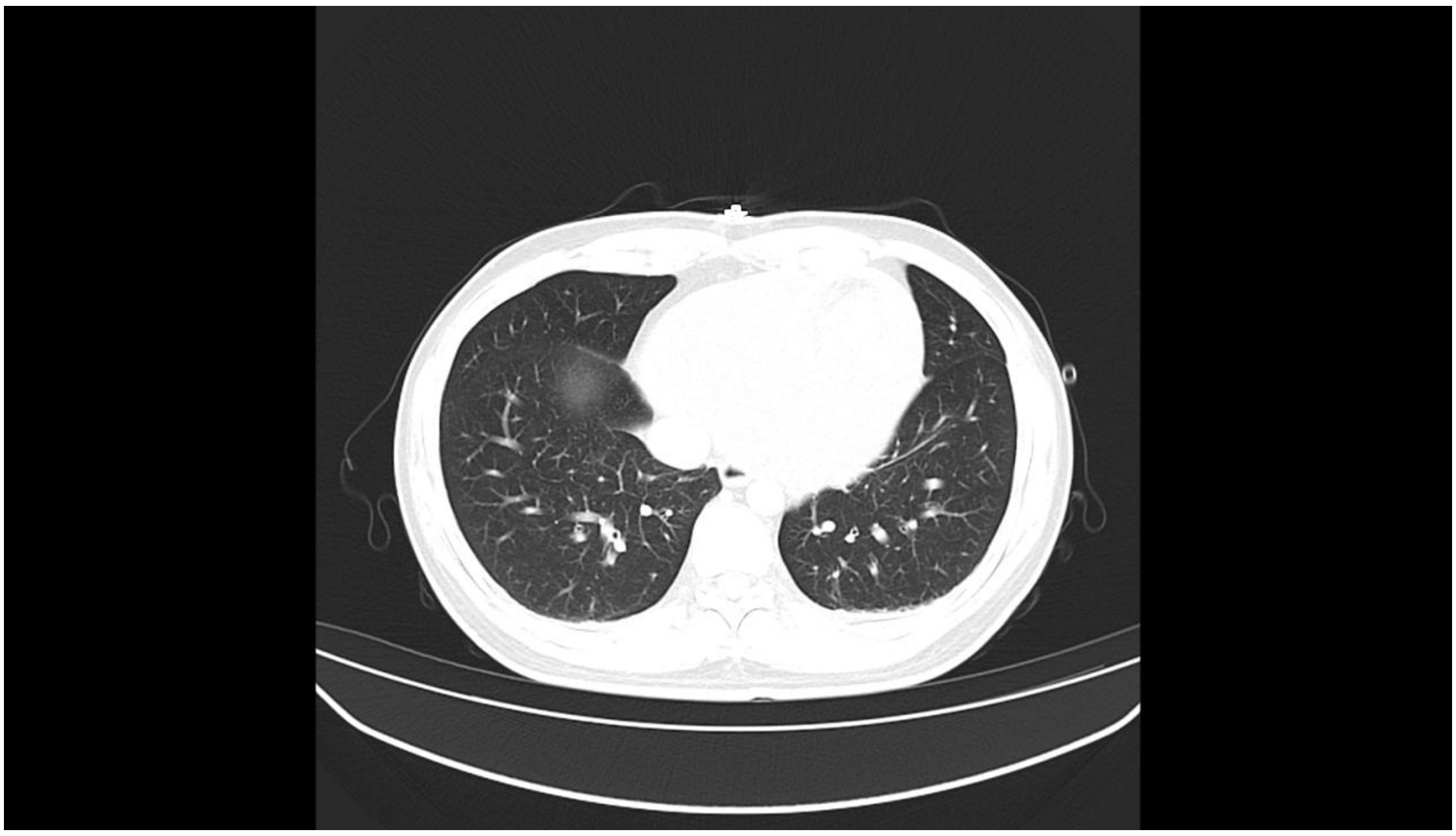

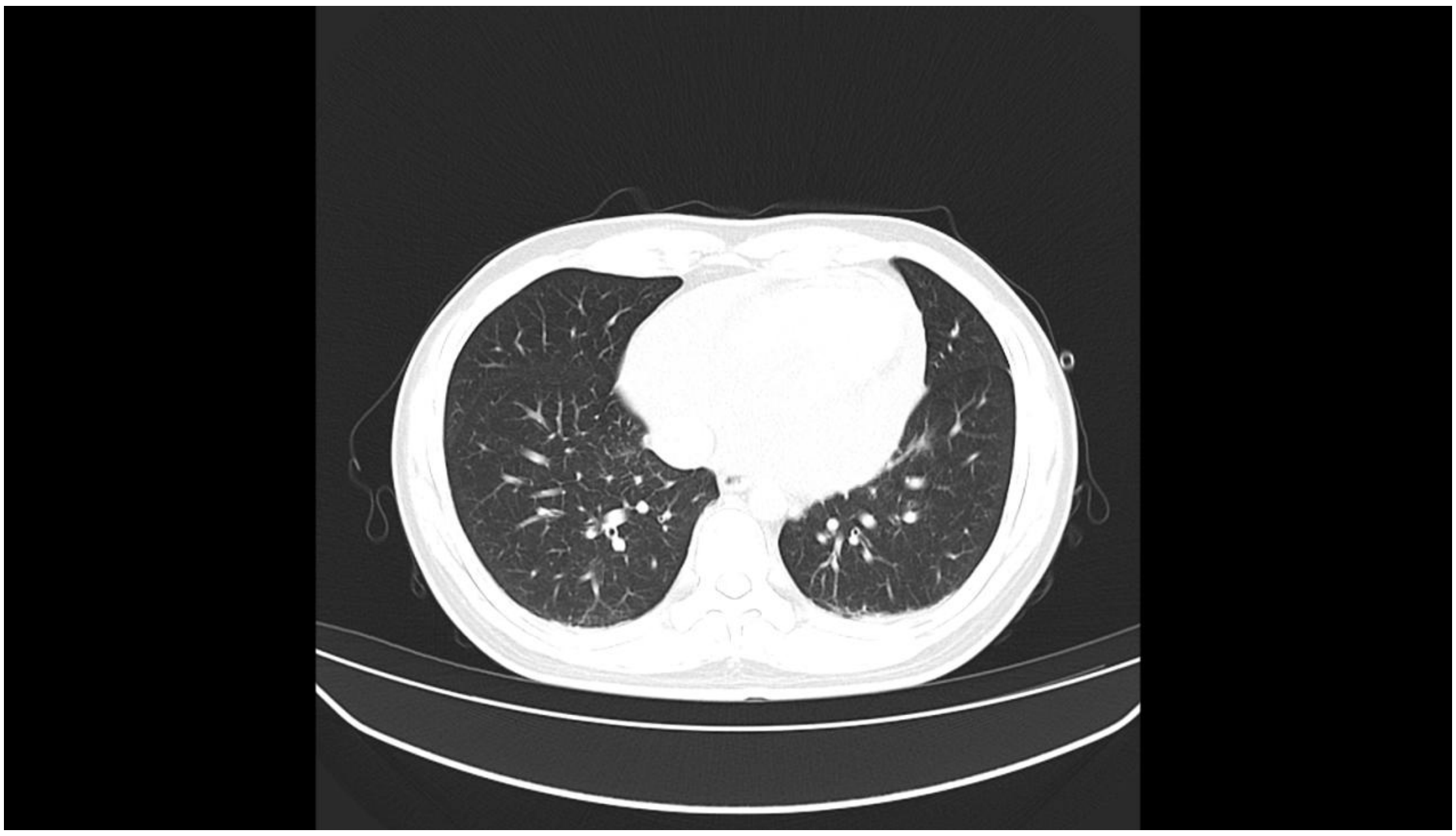

Supplement: Supplementary file 1 [file Datasheet1.pdf]
